# Spinal Anaesthesia Versus General Anaesthesia for Patients With Tibia Shaft Fractures—A Randomized Controlled Study

**DOI:** 10.1111/aas.70111

**Published:** 2025-08-12

**Authors:** Pasi M. Lehto, Merja A. Vakkala, Iikka P. Lantto, Pasi Ohtonen, Janne H. Liisanantti, Timo I. Kaakinen

**Affiliations:** ^1^ Department of Anaesthesiology Oulu University Hospital Oulu Finland; ^2^ Research Group of Anaesthesiology, Medical Research Unit of Translational Medicine University of Oulu Oulu Finland; ^3^ Oulu University Hospital Department of Orthopedics and Traumatology Oulu Finland; ^4^ Research Group of Orthopedic Surgery, Medical Research Unit of Translational Medicine University of Oulu Oulu Finland; ^5^ Research Service Unit Oulu University Hospital Oulu Finland; ^6^ OYS Heart Oulu University Hospital Oulu Finland

**Keywords:** acute compartment syndrome, compartment pressure, delta pressure, general anaesthesia, near‐infrared spectroscopy, spinal anaesthesia, tibia fractures

## Abstract

**Background:**

Concerns about the delayed diagnosis of acute compartment syndrome have led to recommendations favouring general anaesthesia over spinal anaesthesia in surgeries for diaphyseal tibia fractures. However, there is a lack of supporting clinical evidence. This study compared spinal anaesthesia and general anaesthesia in terms of compartment pressures, risk of acute compartment syndrome, and postoperative outcomes in tibia shaft fractures treated with intramedullary nailing.

**Methods:**

A randomized controlled study was carried out at a tertiary hospital from 2011 to 2021. Fifty patients with unilateral tibia shaft fractures were randomly assigned to receive either spinal or general anaesthesia. The primary outcome was compartment and delta pressures in the anterior tibial muscle compartment for 24 h after surgery. Secondary outcomes included near‐infrared spectroscopy values, pain scores, and opioid consumption.

**Results:**

Delta pressures were higher in the spinal anaesthesia group (estimated average effect over 24 h: 6.4 mmHg [CI 0.2–12.6]; *p* = 0.042). However, absolute compartment pressures were comparable between groups (effect estimate: −0.9 mmHg [CI −6.7 to 5.0]; *p* = 0.77). No cases of acute compartment syndrome occurred in the spinal anaesthesia group, while three patients treated with general anaesthesia required fasciotomy. There was no statistical difference in compartment surface oxygenation measured with near‐infrared spectroscopy, pain scores, or median total opioid consumption between the study groups during the 24‐h postoperative follow‐up.

**Conclusion:**

Spinal anaesthesia was not associated with higher compartment pressures compared to general anaesthesia. These findings suggest that prevailing concerns and recommendations about spinal anaesthesia for tibia shaft fracture surgery may need reconsideration and challenge recommendations favouring general anaesthesia as the primary method.

**Editorial Comment:**

This study addresses whether or not spinal anaesthesia might affect acute compartment syndrome and outcomes in tibial shaft fractures. Despite small sample sizes, the findings suggest that spinal anaesthesia does not increase compartment pressures or delay diagnosis of acute compartment syndrome when patients are appropriately monitored. These results support spinal anaesthesia as a safe alternative in selected trauma patients in this context.

**Trial Registration:** Clinicaltrials.gov identifier: NCT01795287

## 1 Introduction

Fractures of tibia diaphysis are associated with a significant risk of developing acute compartment syndrome (ACS). After long bone fractures, 36%–77% of all compartment syndromes are associated with tibia fractures, and the risk of developing ACS following tibia fractures varies, with estimates ranging from 1.5% to 9% [1, 2, 3, 4, 5, 6, 7]. In a recent large cohort study of 26,537 patients with long bone fractures, the incidence of ACS was only 0.1%. Of those diagnosed with ACS, 77.7% were attributed to tibia fractures [8]. Additionally, among those who develop ACS after a tibia fracture, up to 50% are associated with diaphyseal tibia fractures [3].

The standard treatment for compartment syndrome is fasciotomy, a surgical procedure that involves opening the affected muscle compartments to relieve pressure. If left undiagnosed or untreated, ACS can lead to severe complications such as muscle necrosis, nerve damage, infection, and delayed recovery; in severe cases, it may result in kidney failure, multi‐organ failure, or even limb loss [7, 9, 10].

Traditionally, concerns have been raised that effective pain relief provided by regional anesthetics might mask symptoms of ACS, potentially delaying diagnosis and treatment. However, current evidence suggests that regional anesthetics do not obscure the pain associated with compartment syndrome if the patient is adequately monitored [8, 10, 11, 12]. Spinal anaesthesia (SA) induces complete sensory and motor blockade for surgical procedures. It is widely understood that ischemic pain, such as that associated with ACS, is transmitted centrally via sympathetic nerves. Consequently, the sympathectomy resulting from neuraxial blockades, such as SA, can mask ischemic pain during its duration, although not entirely. This prolonged effect of SA has raised concerns about an increased risk of delayed diagnosis of ACS. As a result, it has been generally recommended that patients with tibia fractures should be treated under general anaesthesia (GA) and that SA should be avoided due to concerns it might mask the symptoms of ACS and delay the diagnosis [13, 14]. Modern SA rarely poses this risk because it is designed to last just beyond the duration of surgery. However, while effectively managing pain, it is also important to monitor for signs of ACS, especially in high‐risk cases. No randomized comparative studies have evaluated the safety of regional anesthetics specifically in patients with tibia fractures.

The study aimed to compare SA and GA in terms of recognizing and developing compartment syndrome symptoms in the leg muscles and to assess whether compartment pressures differ between the two anaesthesia methods. The hypothesis was that SA and GA demonstrate comparable safety concerning the risk of developing and recognizing ACS. Secondary outcomes included postoperative pain levels, opioid consumption within the first 24 h after surgery, and near‐infrared spectroscopy (NIRS) values from two different locations in the fractured leg.

## 2 Material and Methods

The study was conducted from 2011 to 2021 at Oulu University Hospital, a tertiary referral hospital serving approximately 737,000 people. The hospital administration and the regional ethics committee of the North Ostrobothnia Hospital District approved the study protocol (diary numbers 125/2011 and 52/2011). Written informed consent was obtained from each participant in the study. The study was registered with clinicaltrials.gov (NCT01795287).

All patients recruited for this study were Finnish‐speaking and over 18 years old. The inclusion criteria were a unilateral tibia shaft fracture requiring surgery, which was intended to be treated with intramedullary nailing. Patients with proximal or distal tibia fractures were excluded from the study.

Exclusion criteria were patient refusal, contraindications to SA (e.g., medications that slow blood clotting or patient‐related coagulopathy, signs of infection at the injection site), severe infection or sepsis, hypovolemia, aortic stenosis, massive obesity, severe generalized atherosclerosis, development of suspected compartment syndrome preoperatively, patients with bilateral tibia fractures or multiple injuries, and patients who did not comprehend the information provided (due to foreign language barriers, dementia, or developmental disabilities). Pregnant patients were not included in the study. Additionally, patients were excluded if the treating anesthesiologists strongly preferred GA over SA.

The patients who met the inclusion criteria were interviewed and informed about the study protocol. Written information was provided before the patients agreed to participate in the study. Those who met the inclusion criteria and consented to participate were randomized into the GA group (*n* = 25) or the SA group (*n* = 25). The investigators of the present study enrolled the participants but did not take part in the treatment of the study patients in the operating theater. The flow chart of the enrollment and allocations is shown in Chart 1.

### 2.1 Anaesthesia Management

The induction of GA was achieved using propofol and fentanyl, with rocuronium administered as a muscle relaxant to facilitate intubation. GA was maintained with sevoflurane and boluses of fentanyl, while additional rocuronium boluses were provided as a muscle relaxant if necessary.

SA was administered according to standard practices. The anesthetic used was bupivacaine hydrochloride, with a dose of 10–15 mg of bupivacaine, depending on the patient's characteristics and estimated time of surgery. This amount of bupivacaine is typically sufficient to maintain anaesthesia of the lower body for 2–4 h, which is an adequate duration for performing surgery on a tibia shaft fracture. Additionally, the use of single‐shot intrathecal fentanyl was permitted at the discretion of the attending anesthesiologist.

### 2.2 Study Methodology Regarding the Primary Outcome

The absolute anterior tibial compartment pressures of the operated lower leg were monitored 24 h postoperatively. At the end of the surgery, a special catheter, the Stryker Intra‐Compartmental Indwelling Slit Catheter (Stryker, Kalamazoo, MI, USA), was inserted into the anterior tibial muscle compartment of the leg operated on by the orthopedic surgeon for continuous measurement of muscle compartment pressure. The catheter was inserted into the muscle compartment at a 45° angle, with the tip positioned approximately 5 cm within the main injury site, which included the fracture line and deeper parts of the muscle compartment [15, 16, 17]. After insertion, the catheter was connected to the arterial line manometer, filled with saline, and checked for air bubbles. To prevent thrombus formation, the line and catheter tip were continuously flushed with 0.9% saline at a rate of 0.1 mL/h. The length of the line was minimized to avoid kinking. The transducer was positioned at the level of the operated leg, displaying continuous pressure readings on the patient monitor. The delta pressure of the anterior tibial compartment was calculated from the difference between diastolic blood pressure and the measured absolute compartment pressure. Delta pressure was used to diagnose potential ACS. Differences in compartment pressures between the fractured and unaffected lower leg were not measured.

The regional oxygen supply of the operated lower leg muscles was continuously measured transcutaneously using NIRS for 24 h postoperatively. NIRS is a non‐invasive monitoring method commonly used in cardiac, aortic, and carotid surgery to monitor the blood supply in the brain [18, 19]. Additionally, NIRS has been used to monitor tissue oxygenation in the lower leg after tibia fractures and in patients with ACS [20, 21]. This study utilized the INVOS (Somanetics Corporation, Troy, Michigan, USA). Sensors were placed on the skin on the posteromedial side and the upper anterior portion of the anterior tibial muscle of the fractured leg. The skin was shaved and cleaned before placing the sensor patches. Control values from the uninjured leg were not recorded.

### 2.3 Post‐Operative Follow‐Up

After the operation, patients were closely monitored in the post‐anaesthesia care unit (PACU) for 24 h. During this period, a nurse conducted a clinical examination of the patients. This examination included checks for leg muscle stiffness, swelling, skin sensation, and peripheral blood circulation (temperature and pulses). The parameters recorded during the PACU stay included absolute compartment pressure, blood pressure readings, calculated compartment delta pressure, and tissue oxygenation monitoring of the anterior and posterior compartments of the operated lower leg. Additionally, postoperative pain was measured using a numeric rating scale (NRS), along with the cumulative opioid consumption calculated in intravenous equivalents of oxycodone. The recorded values were documented every hour for the first 6 h postoperatively, and then every 2 h from the eighth postoperative hour up to 24 h.

In both groups, postoperative pain was managed with 1 g of acetaminophen three times a day, and oxycodone was administered intravenously or orally upon request to achieve NRS values below 4. Anti‐inflammatory pain medication was provided only as needed, at the discretion of the treating anesthesiologist in the PACU. Peripheral nerve blocks were not used for managing postoperative pain.

Additionally, blood samples for myoglobin, creatinine kinase, creatinine, CRP, basic blood count, and tests to evaluate blood coagulation (INR, thrombocytes) were collected. Samples were taken before surgery, after the patient arrived at the PACU, and again at 6 and 24 h after surgery. After 24 h in the PACU, patients were transferred to the ward, and monitoring of the parameters was concluded.

All study patients were followed and rehabilitated according to the hospital's orthopedic guidelines during the postoperative period in the ward and attended the scheduled orthopedic follow‐up appointments 6 weeks, 3 months, and 6 months after discharge. Patient data and recordings were reviewed and evaluated in 2024.

### 2.4 Statistical Analysis

Statistical analyses were performed using SPSS software for Windows (IBM Corp. Released 2021. IBM SPSS Statistics for Windows, Version 28.0. Armonk, NY: IBM Corp). Categorical data are expressed as numbers (*n*) and percentages (%). Continuous variables are presented as medians with the 25th and 75th percentiles. Categorical data were analyzed using Pearson's *χ* ^2^ test or Fisher's exact test, and continuous variables were evaluated using the nonparametric Mann–Whitney *U* test.

The linear mixed model (LMM) was utilized for repeatedly measured continuous data. Time, group, and the Time × Group interaction were set as fixed factors, while patient was included as a random factor in the LMM. Treatment effects are reported with 95% confidence intervals (95% CI) according to the LMM.

No prior comparative data between different anaesthesia methods on compartment pressures after intramedullary nailing of tibia shaft fractures was available in the literature when the study was designed. The power analysis assumed that SA worsens ACS and that the average absolute compartment pressures in the SA group would be 10 mmHg higher than in the GA group. With a standard deviation of 10 mmHg, a significance level of 5%, and a power of 90%, we calculated a total sample size of 44 patients. We then increased the final sample size to 50 patients to account for non‐compliance. This study was powered to compare only the primary endpoint; therefore, the secondary outcomes and subgroup analyses are only hypothesis‐generating.

Patients were randomized into study groups using sealed opaque envelopes to ensure allocation concealment. Two randomization envelopes were employed, each containing 25 slips. The assigned type of anaesthesia was documented on the specific randomization form along with the patient's identification details and date. A separate study form was used during the study period and was subsequently attached to the patient's medical records. Monitoring and follow‐up were performed according to the instructions specified on the study form to maintain consistency and adherence to the study protocol.

## 3 Results

During the study period, 1557 patients with tibia fractures were diagnosed. Of those, 1178 were other than diaphyseal tibia fractures. A total of 379 patients with diaphyseal tibia fractures were identified. Finally, 55 participants were recruited, and data from 50 patients with tibia shaft fractures were analyzed. Of these, 24 were allocated to the SA group, while 26 were assigned to the GA group (Chart 1). The characteristics of both groups were comparable, and no significant differences were observed. The demographics of the study population are presented in Table 1.

**TABLE 1:** Patient characteristics.

	All (*n* = 50)	SA (*n* = 24)	GA (*n* = 26)	*p*
Gender, male	33 (66%)	16 (67%)	17 (65%)	0.92
Age	44.5 [35–56]	46.5 [35–57.5]	42.5 [35–55]	0.34
BMI	25.4 [23–29]	24.6 [22.4–28.4]	27.4 [24.2–30.1]	0.20
ASA	1.5 [1–2]	1 [1–2]	2 [1–2]	0.02
Alcohol related injury	17 (34.0%)	6 (25.0%)	11 (42.3%)	0.20
Smoking	20 (40.0%)	9 (37.5%)	11 (42.3%)	0.78
Diabetes	8 (16.0%)	3 (12.5%)	5 (19.2%)	0.70
HTA	10 (20.0%)	6 (25.0%)	4 (15.4%)	0.49
Side, right	27 (54%)	9 (37.5%)	14 (53.8%)	0.25
AO classification				0.75
42‐A Simple facture	37 (74.0%)	17 (70.8%)	20 (76.9%)	
42‐B Wedge fracture	13 (26.0%)	7 (29.2%)	6 (23.1%)	
Opioids within 12 h pre‐operative, mg iv eqv	5 [2.5–10]	6.75 [2.75–10]	6.25 [5–10.25]	0.58
Opioids 24–48 h post‐operative, mg iv eqv	15 [10–23]	15 [10–20]	20 [11.25–26.25]	0.13
Opioids 48–72 h post‐operative, mg iv eqv	7.5 [3–15] *n* = 44	6 [0.5–13.5] *n* = 24	10 [5–17] *n* = 20	0.24
Waiting time from injury to operation, hours	26 [18–47]	22 [16–48]	33 [19–47]	0.26
Length of stays, days from operation.	4 [3–5]	3 [3–6]	4 [4–5]	0.15
Anaesthesia time, min	154 [130–182]	160 [140–225]	145 [119–180]	0.27
Operation time, min	106 [82–142]	117 [101–171]	89 [75–123]	0.03
Blood pressure during anaesthesia				
MAP low, mmHg	65 [60–73]	69 [65–79]	62 [58–66]	0.001
MAP high, mmHg	94 [81–107]	91 [81–102]	96 [80–112]	0.64
Recorded blood loss during surgical procedure, mL	100 [50–200]	175 [100–275]	100 [50–200]	0.19
GFR 24 h post‐operative	112 [102–121]	116 [103–122]	111 [102–121]	0.65
CK, U/L				
Pre op	255 [159–378]	252 [177–357]	271 [159–378]	0.76
6 h post op	321 [217–530]	291 [216–645]	331 [208–493]	0.99
24 h post op	298 [189–555]	266 [179–583]	319 [195–524]	0.84
Myoglobin, μg/L				
pre op	74 [46–117]	74 [52–117]	76 [44–118]	0.63
6 h post op	121 [81–184]	131 [65–262]	103 [81–179]	0.39
24 h post op	65 [38–108]	79 [39–135]	64 [37–97]	0.51
Fasciotomy, *n* (%)	3 (6%)	—	3 (11.5%)	

*Note:* Values shown in numbers (*n*) and percentages (%), or medians and 25th—75th percentiles.

### 3.1 Tibial Compartment‐Related Results

**FIGURE 1:**
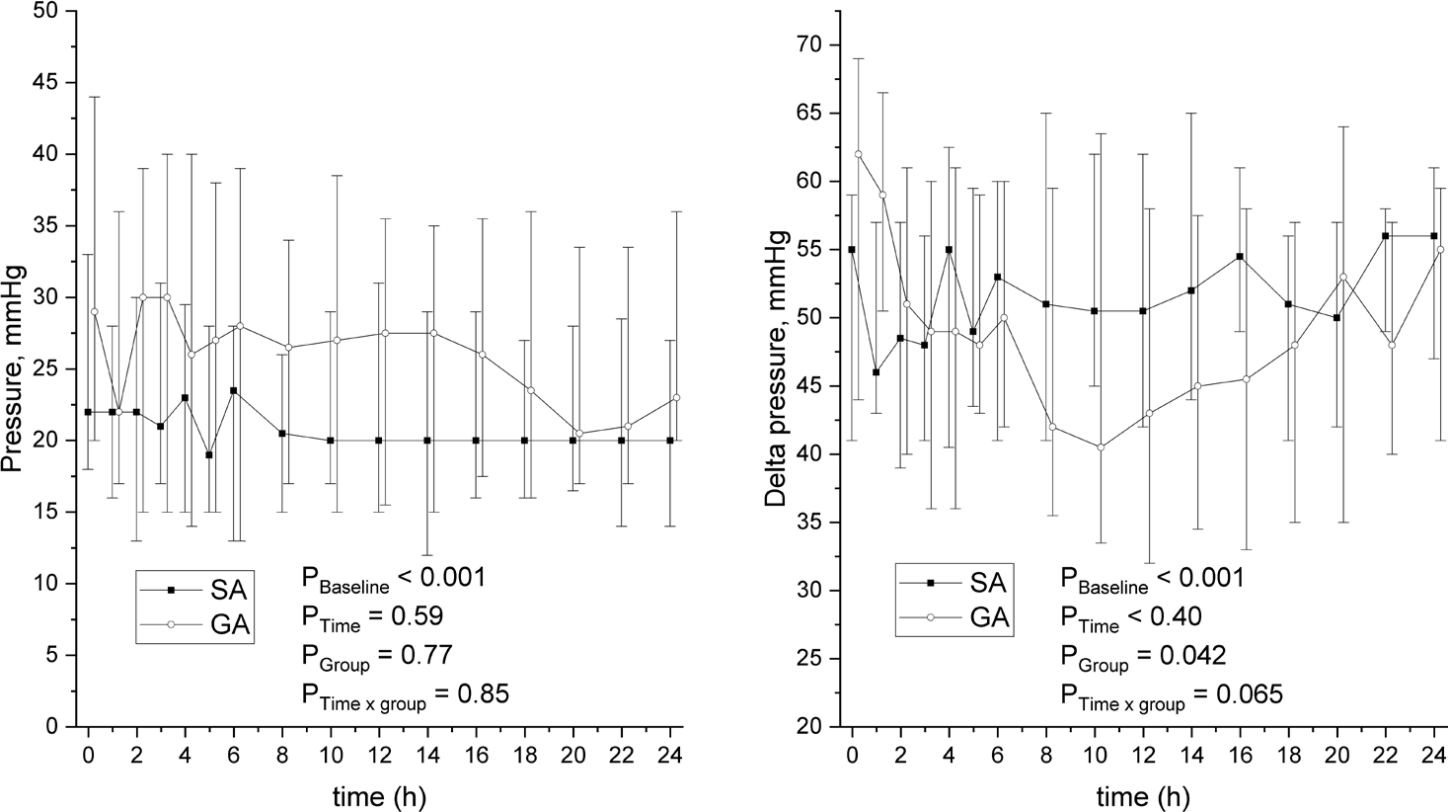
Median absolute compartment pressures and delta pressures in patients treated with spinal anaesthesia (SA) or general anaesthesia (GA). Time 0 = arrival to post anaesthesia care unit.

**FIGURE 2:**
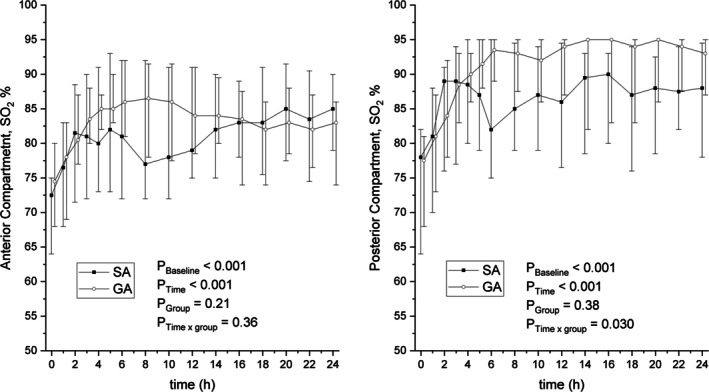
Surface oxygenation values of the anterior and posterior tibial muscle compartments, measured with near‐infrared spectroscopy (NIRS), in patients treated with spinal anaesthesia (SA) or general anaesthesia (GA). Time 0 = arrival to post anaesthesia care unit.

The main finding indicated no difference in absolute compartment pressures between patients treated with SA or GA. The mean compartment pressures (mmHg) during the 24‐h follow‐up were slightly, but not significantly, lower in the SA group at all measured time points (estimate of effect: −0.9 mmHg [CI −6.7 to 5.0]; *p* = 0.77). However, a significant difference was noted in delta pressures between the study groups, with delta pressure being higher in the SA group (estimate of effect: 6.4 mmHg [CI 0.2–12.6]; *p* = 0.042). Absolute compartment pressure (mmHg) and delta pressure during the 24‐h postoperative follow‐up are presented in Figure 1. No statistical difference was observed in compartment surface oxygenation measured with NIRS. During the 24‐h follow‐up, approximately 75% of the recorded NIRS values from the anterior compartment were usable or recorded over the entire 24‐h period in the SA group, compared to 69% in the GA group. For the posterior compartment, 63% of the SA group and 65% of the GA group had usable values. The results concerning surface oxygenation between the study groups in the anterior and posterior compartments are shown in Figure 2. The suprapatellar nailing technique was employed to insert intramedullary nails for 12 patients (SA 7 vs. GA 5) out of 50, with the first surgical procedure utilizing this technique performed in 2016.

### 3.2 Patient‐Related Results

The subjective experience of pain varied significantly between groups during the first two postoperative hours, with patients in the SA group reporting almost no pain on the NRS. After the second postoperative hour and up to 10 h, the patients in the SA group reported higher pain scores compared to the GA group. This difference in reported NRS pain scores between groups was no longer apparent after 12 postoperative hours. Additionally, there were no statistically significant differences in NRS scores between the study groups during the 24‐h postoperative follow‐up (Figure S1).

There was no difference in median total opioid consumption between the study groups during the 24‐h postoperative follow‐up (Figure S2). Mean oxycodone consumption, expressed in equivalent intravenous doses, was significantly lower in the SA group during the first 6 postoperative hours (effect estimate: –6.8 [CI –11.3 to −2.3]; *p* = 0.004). There was no statistical difference in opioid consumption during the 24–72‐h postoperative follow‐up. However, total opioid consumption was slightly higher in the GA group in the ward (Table 1). Both study groups received equal amounts of opioids 12 h preoperatively (Table 1). Preoperative NSAIDs were administered to nine patients in the SA group and 24 patients in the GA group (17 SA vs. 7 GA; *p* = 0.02) during the 24‐h postoperative follow‐up. Preoperative acetaminophen 1000 mg was given to 48 of the 50 study patients; acetaminophen was routinely administered to all but one during the postoperative follow‐up. None of the study patients were habitual opioid users or had received opioids prior to the tibia fracture. In the SA group, the median dose of bupivacaine hydrochloride (5 mg/mL) was 13 mg [12, 13, 14, 15], and intrathecal fentanyl was administered to 11 out of 24 patients with a median dose of 20 μg [20, 21, 22, 23, 24, 25]. Subgroup analysis of pain scores and compartment pressure variables demonstrated no statistically or clinically significant differences between patients receiving intrathecal fentanyl and those not receiving intrathecal fentanyl.

**FIGURE 3:**
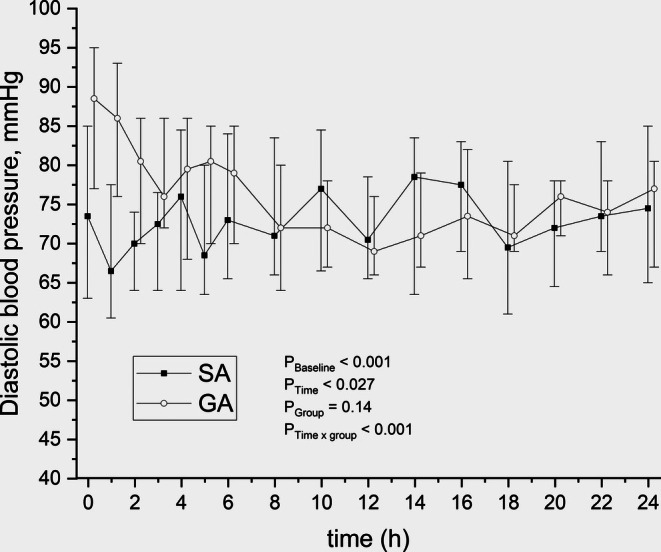
Median diastolic blood pressure in patients treated with spinal anaesthesia (SA) or general anaesthesia (GA). Time 0 = arrival to post‐anaesthesia care unit.

The lowest measured mean arterial pressures (MAP) during surgery were lower in the GA group than in the SA group (Table 1). There was no significant difference in mean arterial pressures (MAP; mmHg) between the SA and GA groups during the 24‐h postoperative follow‐up (effect estimate: 4.42 mmHg [CI –1.53 to 10.38]; *p* = 0.14). The MAPs were lower in the SA group up to 6 h postoperatively and were slightly higher or remained equal between 8 to 24 h postoperatively, with approximate median MAP values of 95 ± 5 mmHg in both groups during the 24‐h follow‐up. No statistical differences in diastolic blood pressures were observed during the 24‐h follow‐up (Figure 3). There were no differences in the quantities of intravenous fluids (crystalloids) administered during the surgical procedure (SA 700 [500–1000] vs. GA 800 [500–1000]; *p* = 0.25). Total intravenous fluids (mL) administered during the surgical procedure and the 24‐h follow‐up in PACU were SA 3000 [2500–3400] versus GA 2500 [1900–4000]; *p* = 0.24. None of the study patients received red blood cells during the surgical procedure or the 24‐h postoperative follow‐up, and there was no difference in the recorded blood loss during the surgical procedure. The duration of the surgical operation was longer in the SA group (Table 1). Among all the study patients, 17 were under the influence of alcohol at the time the tibia fracture occurred.

All study patients had normal postoperative follow‐up visits after discharge. No records in the patient data indicated that the study patients developed complications or later signs associated with high compartment pressure or missed ACS during their hospital recovery. Six patients experienced complications in surgical wound recovery and required subsequent reoperations.

### 3.3 Fasciotomy Patients

Three patients underwent fasciotomy due to signs of ACS, all treated with GA. An orthopedic surgeon made the decision for fasciotomy based on clinical signs and the measured tibial compartment delta pressures, as well as the trends in these pressures. Notable clinical signs included significant pain, muscle compartment stiffness during examination, and, in one case, paresthesia, pallor, and differences in peripheral temperature between the legs. The median delta pressure in the anterior compartment was approximately 20 mmHg lower in the fasciotomy patients compared to other study participants. In these patients, the delta pressures ultimately demonstrated a decreasing trend. In two cases, significantly reduced delta pressures (with the lowest recorded values being 19 and 14 mmHg) and clinical symptoms led to the decision for fasciotomy. One patient exhibited fluctuating delta pressures both above and below 30 mmHg during the follow‐up period; eventually, the clinical presentation combined with the declining pressure trend resulted in a fasciotomy. NIRS values of the anterior compartment were either unmeasurable or unavailable for two patients, while one patient had values ranging from 52% to 64%.

The average time from injury to fasciotomy was 36, and 20 h from the start of anaesthesia. According to the AO/OTA Fracture and Dislocation Classification [22], all fasciotomy patients had tibia shaft fractures classified as 42‐A (simple fractures). The waiting time from injury to surgery was slightly shorter than that of the overall study population (median 18 vs. 26 h). The median duration of the surgical procedure was 85 min. The lowest recorded MAP during anaesthesia was a median of 58 mmHg, while the highest was 95 mmHg. One patient underwent a suprapatellar nailing technique. The length of hospital stays was approximately 18 days.

## 4 Discussion

This study aimed to compare the effects of SA and GA on the compartment pressures and the development of ACS in patients with tibial shaft fractures treated with intramedullary nailing. According to the findings of this study, there is no association between the type of anaesthesia (SA or GA) and absolute compartment pressures in the anterior tibial muscle compartment. However, higher delta pressures were observed in the SA group. Our findings suggest that the safety outcomes related to ACS and compartment pressures are equivalent between SA and GA. Furthermore, SA is not associated with an increased risk of delayed ACS diagnosis when appropriate monitoring is applied. To the best of our knowledge, this is the first randomized controlled study comparing these two methods of anaesthesia in patients with tibial fractures treated with intramedullary nailing.

When the present study was launched, the general recommendation was that patients with tibia fractures should undergo GA because regional and neuraxial anaesthesia could mask the symptoms of developing ACS. The Finnish Clinical Practice Guidelines for tibia fractures, published in 2011, recommend GA for intramedullary nailing of tibial diaphyseal fractures [14]. These guidelines refer to a 1998 orthopedic textbook [23] advocating for GA but do not provide any supporting sources for this claim. GA was endorsed in the 2013 third edition due to concerns that SA or regional anaesthesia techniques might obscure ACS symptoms [13]. The 2021 fourth edition maintains GA as the preferred approach in tibia shaft fracture surgery for the same reasons, but the authors acknowledge that this subject is not well studied [24]. An educational article from 2021 [25] warns against the use of regional anaesthesia despite the lack of sufficient research evidence regarding whether regional anaesthesia delays the diagnosis of compartment syndrome [11, 26]. This perspective contradicts current recommendations and studies, which have concluded that SA and other forms of regional anaesthesia are safe for patients with tibia fractures [2, 26, 27, 28, 29]. Furthermore, concerns about the development or missed diagnoses of ACS in patients with tibia shaft fractures after SA are less relevant due to its short‐lived anesthetic and analgesic effects [26], but no previous randomized studies are addressing this.

### 4.1 The Risk of ACS and Intraoperative Considerations

The pathophysiological relationship between compartment pressure, blood flow, and blood pressure is multifactorial. Traumatic tibia fractures and muscle tissue injuries cause swelling, which increases compartment pressure and reduces blood flow, leading to oxygen deprivation. To compensate, arterioles dilate, enhancing blood flow, but impaired venous return disrupts capillary permeability, resulting in fluid buildup and further pressure elevation. Venous return and lymphatic flow are compromised at lower pressures than arterial circulation [7, 9, 10, 30, 31]. Since anaesthesia typically lowers blood pressure, maintaining adequate perfusion is crucial in patients at risk of ACS, as hypotension may accelerate its progression. Neuraxial anaesthesia, such as SA and epidural anaesthesia, alters blood flow due to sympathetic blockade inducing vasodilation, which may increase compartment pressure if tissues cannot accommodate the increased perfusion. Conversely, continuous nerve blocks and vasodilation could potentially benefit the affected extremity by promoting compartment drainage. A recent review indicates that patients without hypertension are more likely to develop ACS [1]. Elevated blood pressure may help counteract rising compartment pressure, preserving the pressure gradient essential for delivering oxygen and nutrients to muscle tissue. In our study, patients in the GA group had lower intraoperative MAP values than those in the SA group, while those treated with SA exhibited more stable intraoperative blood pressures, as shown by digital anaesthesia recordings. Nevertheless, the low blood pressures were brief in both groups and cannot be considered to have impacted postoperative compartment pressures during the 24‐h follow‐up. Furthermore, SA may enhance muscle perfusion, improve venous return, and reduce capillary hydrostatic pressure, potentially mitigating ischemia, fluid accumulation, and swelling compared to GA.

Patient positioning during tibial nailing affects intra‐compartmental pressures [3, 15, 32]. The infrapatellar technique carries risks of increased pressure and ACS due to deep knee flexion and popliteal support that obstructs venous drainage. In contrast, the suprapatellar technique improves alignment and reduces soft tissue complications, as it is conducted with the knee nearly fully extended, thereby minimizing pressure risks. Suprapatellar nailing is associated with fewer compartment syndromes and fasciotomies in cases of tibia shaft fractures [33].

### 4.2 Muscle Compartment Pressures During the Post‐Operative Follow‐Up

In our study, postoperative delta pressures were higher and absolute compartment pressures lower in the SA group; although the difference in absolute compartment pressures between the study groups was not statistically significant. Despite similar diastolic pressures between the SA and GA groups, the higher delta pressures in the SA group suggest a greater perfusion gradient, which may reduce the risk of ACS.

There is inconsistency in the critical thresholds of absolute compartment pressures reported in the literature, ranging from 30 to 50 mmHg. An absolute pressure of 30 mmHg indicates that capillary perfusion becomes inadequate. However, absolute thresholds overlook perfusion pressure, which is sustained by blood pressure [7, 9, 10, 15, 30, 31, 34]. The widely accepted delta pressure (diastolic blood pressure minus compartment pressure) threshold is 30 mmHg, with the risk of ACS increasing if it falls below this value. Low blood pressure further elevates the risk of ACS and muscle damage, although short‐term high compartment pressure spikes may not necessarily indicate ACS. However, the accuracy of delta pressure measurements may be affected by factors such as fluctuations in blood pressure. Therefore, relying solely on direct compartment pressure measurements with existing thresholds may not reliably diagnose ACS [35]. Trend analysis and clinical symptoms are essential for diagnosing ACS, and making decisions regarding fasciotomy [7, 9, 10, 15, 30, 31, 34].

Postoperative compartment pressure may be influenced by several factors, including limb positioning, patient characteristics, and injury severity. Younger patients may be at greater risk because their muscle compartments have limited capacity to expand [3]. Postoperative swelling from surgical trauma or delayed fluid mobilization may contribute to increased compartment pressures. However, there were no statistical differences in patient characteristics or postoperative treatment between groups in our study.

### 4.3 The Role of Near‐Infrared Spectroscopy (NIRS) in ACS Detection

Our study found no significant differences in NIRS values between the SA and GA groups, suggesting that SA does not impair lower leg perfusion. The values in the anterior compartment of the SA group were comparable to previous studies that reported average oxygenation values ranging from 69% to 82% ± 4% in uninjured compartments [36, 37]. It is noted that ischemic compartments demonstrate a reduction in NIRS values in injured limbs [21], and compromised compartments exhibit values of 50%–56% ± 16%–27% before fasciotomy [36, 37]. In our study, one patient with fasciotomy presented consistently lower oxygenation values without any decline before fasciotomy. The two other fasciotomy patients had no measurable values.

Usable NIRS values were successfully recorded until the end of the 24‐h monitoring period in most cases for both the anterior and posterior compartments across both groups. This contrasts with a previous study where only 31.6% of patients had useful data from NIRS, along with available data for just 9.1% of the expected monitoring time [37]. Nonetheless, no conclusions can be made about the usefulness of NIRS in our series.

An interesting finding was that NIRS values were notably high in both groups, particularly in the posterior compartment of the GA group, with values reaching 95% or higher. It can be speculated that this indicates hyperemia and impaired oxygen extraction in the tissue due to elevated compartment pressure. NIRS has been observed to detect initial hyperemia following a traumatic event, resulting in increased tissue oxygenation [20, 37, 38, 39]. In cases of tibia fractures, a tissue oxygenation increase of approximately 15% has been reported using NIRS [20]. Additionally, in severe lower leg injuries, elevated NIRS values have been noted compared to the contralateral uninjured limb, although this difference diminishes as compartment pressure rises, causing ischemia [36, 37]. Most likely in the present study, the high NIRS values, especially regarding surface oxygenation in the posterior compartment, can be attributed to measurement errors or surface blood flow, as NIRS has a limited penetration depth of approximately 3 cm.

NIRS has been suggested as a potential tool for the early detection of ACS, as declining tissue oxygenation may indicate compromised blood flow due to increased compartment pressures [20, 21]. However, it remains unclear whether NIRS alone can reliably diagnose ACS; previous studies have shown mixed results regarding the availability of useful values, along with its sensitivity and specificity. Caution and clinical suspicion are advised when using NIRS to monitor ACS.

### 4.4 Postoperative Pain Sensation and Opioid Consumption

The SA group experienced more pain during the second and tenth postoperative hours, as assessed by the NRS, with values reflecting the highest reported pain during the follow‐up periods and corresponding with the administered opioid doses. However, throughout the entire 24‐h follow‐up, no significant differences were found in NRS scores. The total amount of oxycodone was lower at all measured intervals in the SA group during the initial 6 h postoperatively, which can be attributed to the ongoing analgesic effect of SA and, to some degree, the effect of intrathecal fentanyl [40]. Between 8 and 24 h postoperatively, no significant difference in cumulative opioid consumption was observed between the study groups.

In the three diagnosed cases of ACS, significant pain and muscle stiffness were the primary symptoms, with an average duration of 36 h from injury to fasciotomy, highlighting the need for vigilant monitoring. Severe pain that does not respond to standard analgesics and opioids is the key indicator of ACS [3, 15].

The ability of opioids to provide effective analgesia for ischemic pain has raised concerns about delayed diagnosis of ACS in high‐risk patients. Moreover, postoperative opioid analgesia, particularly morphine, has been suspected of masking pain related to ACS [41, 42, 43]. However, these case reports often lack adequate postoperative follow‐up in high‐risk patients, which limits definitive conclusions. In this study, opioid use did not differ between the groups, and no association can be drawn between opioid consumption and the potential masking of ACS symptoms; we did not find any indications of potentially missed ACS symptoms based on patient recordings made after discharge.

Pain signals are transmitted to the spinal cord through fast‐conducting myelinated A‐type fibers and slow‐conducting unmyelinated C‐fibers. Ischemic pain, such as that seen in ACS, is primarily conducted via C‐fibers. Despite adequate motor and sensory blockade from SA, ischemic pain may still be perceived, as C‐fibers recover earlier than A‐type fibers [44]. As A‐type fiber activity returns after SA wears off, ischemic pain is expected to become detectable. The addition of intrathecal opioids reduces ischemic pain [45]. In our study, the median dose of intrathecal fentanyl may have provided analgesia for up to 4 h [46], without prolonging motor block [47]. However, less than half of the patients in the SA group received intrathecal fentanyl. We do not consider SA or intrathecal fentanyl a clinically relevant risk for delayed ACS diagnosis.

## 5 Limitations

Our study has several limitations. First, the sample size was relatively small (*n* = 50), which restricts our ability to detect minor differences between groups. Second, there are no randomized controlled trials comparing SA and GA in the context of ACS risk, making direct comparisons challenging. The primary aim was not to establish equivalence or non‐inferiority, but rather to determine whether a significant difference existed in compartment pressures between study groups. Although the results may suggest equivalence, they should be interpreted with caution because the sample size was calculated under the assumption that a significant difference between the groups exists. Therefore, a non‐significant finding in compartment pressures alone cannot justify the conclusion that there is no difference. Furthermore, the study was not adequately powered for secondary outcomes. Third, we did not use a computerized randomization technique. One limitation of this study is the imbalance in group sizes caused by missing data, which we noticed during the data collection phase when it was no longer possible to recruit additional participants. The initial sample size calculation indicated that fewer participants would have been sufficient to meet the study objectives, but the sample was increased to account for potential data loss. Therefore, despite the imbalance, the total sample size exceeded the required minimum, making any impact on the study's findings unlikely. Additionally, a review of patient records confirmed that those excluded because of missing data did not develop ACS.

The lack of standardization in the use of intrathecal fentanyl is also a limitation of this study, as it may potentially impact postoperative pain and pressure responses in patients at risk of ACS. However, no statistically or clinically significant differences were observed in pain scores or pressure variables between patients who received intrathecal fentanyl and those who did not. Still, the study population in this subgroup is too small to draw any conclusions about intrathecal fentanyl.

Our study took a long time to complete. Nevertheless, during this period, no notable changes were observed in the overall management of patients with tibial diaphyseal fractures in our hospital or nationally [48]. The use of suprapatellar techniques has increased in recent years, which may influence compartment pressures and could act as a confounding factor. However, even though this technique may have affected outcomes, its impact would likely have been more pronounced had it been used more frequently or assigned to only one of the groups. There may have been variations in the needle placement technique for pressure measurement of the anterior compartment due to different operating surgeons. The surgeon may also have affected the duration of the surgery, but this is considered to have been evenly distributed among the study groups.

Recruiting participants for the study proved somewhat challenging, particularly due to the lengthy follow‐up protocol, which some patients viewed negatively. Another significant factor was patients' existing preference for a specific type of anaesthesia. Moreover, the final phase of the study coincided with the COVID‐19 pandemic, during which clinical follow‐up studies in our surgical department were largely suspended; resources were redirected to other areas of patient care.

Still, this study provides important clinical insights into the safety of SA in tibia fracture surgery. We found no evidence that SA raises compartment pressure or increases the risk of ACS. Our findings challenge the traditional recommendations favouring GA over SA for tibia fracture patients due to concerns about ACS.

## 6 Conclusion

Spinal anaesthesia was not associated with increased postoperative compartment pressures compared to general anaesthesia. These results indicate that spinal anaesthesia could be a feasible alternative to general anaesthesia for tibia shaft fracture surgery.

## Author Contributions

P.M.L.: conceptualization, methodology, validation, investigation, formal analysis, writing original draft, writing – review and editing, data curation. M.A.V.: conceptualization, investigation, methodology, validation, writing – review and editing, supervision. I.P.L.: conceptualization, methodology, validation, writing – review and editing. P.O.: validation, formal analysis, data curation, writing – review and editing. J.H.L.: conceptualization, validation, writing – review and editing. T.I.K.: conceptualization, methodology, investigation, validation, formal analysis, data curation, writing – review and editing, supervision.

## Conflicts of Interest

The authors declare no conflicts of interest.

## Supporting information


**Figure S1:** Postoperative pain scores between spinal anaesthesia (SA) and general anaesthesia (GA) groups measured with a numeric rating scale (NRS). Time 0 = arrival to post anaesthesia care unit.

**Figure S2:** Total mean cumulative postoperative opioid consumption in milligrams of intravenous oxycodone equivalents within 24 h between the SA and GA groups. Time 0 = arrival to post anaesthesia care unit.

## Data Availability

The data that support the findings of this study are available on request from the corresponding author. The data are not publicly available due to privacy or ethical restrictions.

## References

[1] T. Wang , J. Guo , Y. Long , and Z. Hou , “Predictors of Acute Compartment Syndrome in Patients With Tibial Fractures: A Meta‐Analysis,” International Orthopaedics 47 (2023): 51–65, 10.1007/s00264-022-05643-3. 36450888

[2] N. Hilber , A. Dodi , S. Blumenthal , H. Bruppacher , A. Borgeat , and J. Aguirre , “The Impact of Regional Anaesthesia in Masking Acute Compartment Syndrome After Limb Trauma,” Journal of Clinical Medicine 20, no. 13 (2024): 1787, 10.3390/jcm13061787. PMC10971118 38542011

[3] B. Shadgan , G. Pereira , M. Menon , S. Jafari , W. D. Reid , and P. J. O'Brien , “Risk Factors for Acute Compartment Syndrome of the Leg Associated With Tibial Diaphyseal Fractures in Adults,” Journal of Orthopaedics and Traumatology 16 (2015): 185–192, 10.1007/s10195-014-0330-y. 25543232 PMC4559534

[4] S. Park , J. Ahn , A. O. Gee , A. F. Kuntz , and J. L. Esterhai , “Compartment Syndrome in Tibial Fractures,” Journal of Orthopaedic Trauma 23 (2009): 514–518, 10.1097/BOT.0b013e3181a2815a. 19633461

[5] M. M. McQueen , P. Gaston , and C. M. Court‐Brown , “Acute Compartment Syndrome. Who Is at Risk?,” Journal of Bone and Joint Surgery (British Volume) 82 (2000): 200–203, 10.1302/0301-620X.82B2.9799. 10755426

[6] M. M. McQueen , J. Christie , and C. M. Court‐Brown , “Acute Compartment Syndrome in Tibial Diaphyseal Fractures,” Journal of Bone and Joint Surgery (British Volume) 78‐B (1996): 95–98. 8898136

[7] A. von Keudell , M. Weaver , and P. Appleton , “Diagnosis and Treatment of Acute Extremity Compartment Syndrome,” Lancet 386 (2015): 1299–1310. 26460664 10.1016/S0140-6736(15)00277-9

[8] S. Chembrovich , B. Ihnatsenka , C. Smith , et al., “Incidence of Acute Compartment Syndrome With Routine Use of Regional Anaesthesia for Patients With Long Bone Fractures: A Large Single‐Center Retrospective Review From a Level I Trauma Tertiary Academic Institution,” Regional Anesthesia and Pain Medicine 49 (2024): 505–510, 10.1136/rapm-2023-104460. 37696649

[9] K. J. Elliott and A. Johnstone , “Diagnosing Acute Compartment Syndrome,” Journal of Bone and Joint Surgery (American Volume) 85 (2003): 625–632. 12892179

[10] S. Mannion and X. Capdevila , “Acute Compartment Syndrome and the Role of Regional Anaesthesia,” International Anesthesiology Clinics 48 (2010): 85–105. 20881529 10.1097/AIA.0b013e3181f1e7de

[11] E. B. S. Driscoll , A. H. Maleki , L. Jahromi , et al., “Regional Anaesthesia or Patient‐Controlled Analgesia and Compartment Syndrome in Orthopedic Surgical Procedures: A Systematic Review,” Regional Anesthesia and Pain Medicine 9 (2016): 65–81, 10.2147/LRA.S109659. PMC5063486 27785097

[12] G. Mar , M. J. Barrington , and B. McGuirk , “Acute Compartment Syndrome of the Lower Limb and the Effect of Postoperative Analgesia on Diagnosis,” British Journal of Anaesthesia 102 (2009): 3–11. 19022795 10.1093/bja/aen330

[13] D. S. Horwitz and E. N. Kubiak , “Chapter 29: Tibial Shaft Fractures: Intramedullary Nailing,” in Fractures Master Techniques in Orthopaedic Surgery, 3rd ed., ed. D. A. Wiss (Lippincott Williams & Wilkins, 2013).

[14] Suomalaisen Lääkäriseuran Duodecimin ja Suomen Ortopediyhdistys ry asettama työryhmä , Säärimurtumat (Suomalainen Lääkäriseura Duodecim, 2011), www.kaypahoito.fi.

[15] S. A. Olson and R. R. Glasgow , “Acute Compartment Syndrome in Lower Extremity Musculoskeletal Trauma,” Journal of the American Academy of Orthopaedic Surgeons 13 (2005): 436–444. 16272268 10.5435/00124635-200511000-00003

[16] M. M. Heckman , T. E. Whitesides, Jr. , S. R. Grewe , and M. D. Rooks , “Compartment Pressure in Association With Closed Tibial Fractures: The Relationship Between Tissue Pressure, Compartment, and the Distance From the Site of the Fracture,” Journal of Bone and Joint Surgery (American Volume) 76 (1994): 1285–1292. 8077257 10.2106/00004623-199409000-00002

[17] M. Nakhostine , J. R. Styf , S. van Leuven , A. R. Hargens , and D. H. Gershuni , “Intramuscular Pressure Varies With Depth: The Tibialis Anterior Muscle Studied in 12 Volunteers,” Acta Orthopaedica Scandinavica 64 (1993): 377–381, 10.3109/17453679308993649. 8322604

[18] J. C. Hirsch , J. R. Charpie , R. G. Ohye , and J. G. Gurney , “Near‐Infrared Spectroscopy: What We Know and What We Need to Know—A Systematic Review of the Congenital Heart Disease Literature,” Journal of Thoracic and Cardiovascular Surgery 137, no. 1 (2009): 154–159, 10.1016/j.jtcvs.2008.08.005. 19154918

[19] K. M. Lanning , L. A. Ylikauma , T. M. Erkinaro , P. P. Ohtonen , M. A. Vakkala , and T. I. Kaakinen , “Changes in Transcranial Near‐Infrared Spectroscopy Values Reflect Changes in Cardiac Index During Cardiac Surgery,” Acta Anaesthesiologica Scandinavica 67 (2023): 599–605, 10.1111/aas.14210. 36740457

[20] M. S. Shuler , W. M. Reisman , T. E. Whitesides, Jr. , et al., “Near‐Infrared Spectroscopy in Lower Extremity Trauma,” Journal of Bone and Joint Surgery (American Volume) 91 (2009): 1360–1368, 10.2106/JBJS.H.00347. 19487513

[21] M. S. Shuler , W. M. Reisman , T. L. Kinsey , et al., “Correlation Between Muscle Oxygenation and Compartment Pressures in Acute Compartment Syndrome of the Leg,” Journal of Bone and Joint Surgery (American Volume) 92 (2010): 863–870, 10.2106/JBJS.I.00816. 20360509

[22] E. G. Meinberg , J. Agel , C. S. Roberts , M. D. Karam , and J. F. Kellam , “Fracture and Dislocation Classification Compendium‐2018,” Journal of Orthopaedic Trauma 32, no. Suppl 1 (2018): S1–S170, 10.1097/BOT.0000000000001063. 29256945

[23] D. A. Wiss , ed., Master Techniques in Orthopaedic Surgery: Fractures, 1st ed. (Lippincott Williams & Wilkins, 1998).

[24] M. D. Riedel and R. V. O'Toole , “Tibial Shaft Fractures: Intramedullary Nailing,” in Master Techniques in Orthopaedic Surgery: Fractures, 4th ed., ed. M. J. Gardner (Wolters Kluwer, 2021), 555–579.

[25] T. Dwyer , D. Burns , A. Nauth , K. Kawam , and R. Brull , “Regional Anaesthesia and Acute Compartment Syndrome: Principles for Practice,” Regional Anesthesia and Pain Medicine 46 (2021): 1091–1099, 10.1136/rapm-2021-102735. 34187911

[26] P. Marhofer , J. Halm , G. C. Feigl , T. Schepers , and M. W. Hollmann , “Regional Anaesthesia and Compartment Syndrome,” Anesthesia and Analgesia 133 (2021): 1348–1352, 10.1213/ANE.0000000000005661. 34255752

[27] M. H. Nathanson , W. Harrop‐Griffiths , D. J. Aldington , et al., “Regional Analgesia for Lower Leg Trauma and the Risk of Acute Compartment Syndrome: Guideline From the Association of Anaesthetists,” Anaesthesia 76, no. 11 (2021): 1518–1525, 10.1111/anae.15504. 34096035 PMC9292897

[28] A. R. Deemer , A. Ganta , P. Leucht , S. Konda , and K. A. Egol , “Regional Anaesthesia Is Safe and Effective for Low‐Energy Tibial Plateau Fractures,” Orthopedics 46, no. 6 (2023): 358–364, 10.3928/01477447-20230407-02. 37052595

[29] A. Ganta , N. D. Fisher , K. Gibbons , et al., “Regional Anaesthesia Is Safe for Use in Intramedullary Nailing of Low‐Energy Tibial Shaft Fractures,” Injury 55, no. 8 (2024): 111636, 10.1016/j.injury.2024.111636. 38870608

[30] C. H. Rorabeck and K. M. Clarke , “The Pathophysiology of the Anterior Tibial Compartment Syndrome: An Experimental Investigation,” Journal of Trauma 18 (1978): 299–304. 660681 10.1097/00005373-197805000-00001

[31] A. H. Schmidt , “Acute Compartment Syndrome,” Injury 48, no. Suppl 1 (2017): S22–S25, 10.1016/j.injury.2017.04.024. 28449851

[32] A. L. Goldsmith and M. I. McCallum , “Compartment Syndrome as a Complication of the Prolonged Use of the Lloyd‐Davies Position,” Anaesthesia 51, no. 11 (1996): 1048–1052, 10.1111/j.1365-2044.1996.tb15003.x. 8943599

[33] E. E. Honkonen , J. P. Repo , H. Lehtokangas , et al., “Suprapatellar Tibial Fracture Nailing Is Associated With Lower Rate for Acute Compartment Syndrome and the Need for Fasciotomy Compared With the Infrapatellar Approach,” Journal of Orthopaedics and Traumatology 25 (2024): 5, 10.1186/s10195-024-00749-3. 38282098 PMC10822828

[34] K. Enneking , L. Le‐Wendling , and B. Ihnatsenka , “Anaesthesia for Orthopedic Trauma,” in UpToDate, ed. R. Maniker and M. Crowley (UpToDate, Inc., 2024), https://www.uptodate.com/contents/anaesthesia‐for‐orthopedic‐trauma.

[35] M. J. Prayson , J. L. Chen , D. Hampers , M. Vogt , J. Fenwick , and R. Meredick , “Baseline Compartment Pressure Measurements in Isolated Lower Extremity Fractures Without Clinical Compartment Syndrome,” Journal of Trauma 60 (2006): 1037–1040, 10.1097/01.ta.0000215444.05928.2f. 16688067

[36] M. S. Shuler , M. Roskosky , T. Kinsey , et al., “Continual Near‐Infrared Spectroscopy Monitoring in the Injured Lower Limb and Acute Compartment Syndrome,” Bone & Joint Journal 100‐B (2018): 787–797, 10.1302/0301-620X.100B6.BJJ-2017-0736.R3. 29855235

[37] A. H. Schmidt , M. J. Bosse , K. P. Frey , et al., “Predicting Acute Compartment Syndrome (PACS): The Role of Continuous Monitoring,” Journal of Orthopaedic Trauma 31 (2017): S40–S47, 10.1097/BOT.0000000000000796. 28323801

[38] W. M. Reisman , M. S. Shuler , M. Roskosky , T. L. Kinsey , and B. A. Freedman , “Use of Near‐Infrared Spectroscopy to Detect Sustained Hyperaemia Following Lower Extremity Trauma,” Military Medicine 181, no. 2 (2016): 111–115, 10.7205/MILMED-D-14-00689. 26837078

[39] M. Novak , M. Penhaker , P. Raska , L. Pleva , and M. Schmidt , “Extremity Compartment Syndrome: A Review With a Focus on Non‐Invasive Methods of Diagnosis,” Frontiers in Bioengineering and Biotechnology 10 (2022): 10, 10.3389/fbioe.2022.801586. PMC9340208 35923576

[40] P. M. Lehto , M. A. Vakkala , S. Alahuhta , et al., “Difference in Postoperative Opioid Consumption After Spinal Versus General Anaesthesia for Ankle Fracture Surgery‐A Retrospective Cohort Study,” Acta Anaesthesiologica Scandinavica 65 (2021): 1109–1115, 10.1111/aas.13845. 33963533

[41] P. Harrington , J. Bunola , A. J. Jennings , D. J. Bush , and R. M. Smith , “Acute Compartment Syndrome Masked by Intravenous Morphine From a Patient‐Controlled Analgesia Pump,” Injury 31 (2000): 387–389. 10775698 10.1016/s0020-1383(99)00308-3

[42] H. Richards , A. Langston , R. Kulkarni , and E. Downes , “Does Patient‐Controlled Analgesia Delay the Diagnosis of Compartment Syndrome Following Intramedullary Nailing of the Tibia?,” Injury 35 (2004): 296–298. 15124799 10.1016/s0020-1383(03)00311-5

[43] Q. Azam , M. Ali , M. Ruwaili , and H. Al Sayed , “Compartment Syndrome Obscured by Post‐Operative Epidural Analgesia,” Clinics and Practice 27, no. 2 (2012): e19. 10.4081/cp.2012.e19 PMC3981334 24765418

[44] A. J. Gissen , B. G. Covino , and J. Gregus , “Differential Sensitivities of Mammalian Nerve Fibers to Local Anesthetic Agents,” Anesthesiology 53, no. 6 (1980): 467–474, 10.1097/00000542-198012000-00006. 7457962

[45] M. Tuominen , H. Valli , E. Kalso , and P. H. Rosenberg , “Efficacy of 0.3 Mg Morphine Intrathecally in Preventing Tourniquet Pain During Spinal Anaesthesia With Hyperbaric Bupivacaine,” Acta Anaesthesiologica Scandinavica 32, no. 2 (1988): 113–116, 10.1111/j.1399-6576.1988.tb02697.x. 2894739

[46] B. M. Bujedo , “A Review of Epidural and Intrathecal Opioids Used in the Management of Postoperative Pain,” Journal of Opioid Management 8, no. 3 (2012): 177–192, 10.5055/jom.2012.0114. 22798178

[47] H. Singh , J. Yang , K. Thornton , and A. H. Giesecke , “Intrathecal Fentanyl Prolongs Sensory Bupivacaine Spinal Block,” Canadian Journal of Anaesthesia 42, no. 11 (1995): 987–991, 10.1007/BF03011070. 8590509

[48] A. A. J. Ylitalo , K. A. Dahl , A. Reito , and E. Ekman , “Changes in Operative Treatment of Tibia Fractures in Finland Between 2000 and 2018: A Nationwide Study,” Scandinavian Journal of Surgery 111 (2022): 65–71, 10.1177/14574969221111612. 36000729

