# Supplementary material for: Spinal Anaesthesia Versus General Anaesthesia for Patients With Tibia Shaft Fractures—A Randomized Controlled Study
Source: Acta Anaesthesiol Scand. 2025 Aug 12;69(8):e70111. doi: 10.1111/aas.70111 (PMC12340734; doi:10.1111/aas.70111)
Supplement: Supplementary file 1 — Figure S1: Postoperative pain scores between spinal anaesthesia (SA) and general anaesthesia (GA) groups measured with a numeric rating scale (NRS). Time 0 = arrival to post anaesthesia care unit. [file AAS-69-0-s002.docx]

Figure S1. Postoperative pain scores between spinal anaesthesia (SA) and general anaesthesia (GA) groups measured with a numeric rating scale (NRS). Time 0 = arrival to post anaesthesia care unit.
