# Supplementary material for: Spinal Anaesthesia Versus General Anaesthesia for Patients With Tibia Shaft Fractures—A Randomized Controlled Study
Source: Acta Anaesthesiol Scand. 2025 Aug 12;69(8):e70111. doi: 10.1111/aas.70111 (PMC12340734; doi:10.1111/aas.70111)
Supplement: Supplementary file 2 — Figure S2: Total mean cumulative postoperative opioid consumption in milligrams of intravenous oxycodone equivalents within 24 h between the SA and GA groups. Time 0 = arrival to post anaesthesia care unit. [file AAS-69-0-s001.docx]

Figure S2. Total mean cumulative postoperative opioid consumption in milligrams of intravenous oxycodone equivalents within 24 hours between the SA and GA groups. Time 0 = arrival to post anaesthesia care unit.
